# A framework for evaluating implementation, impact, and cost-effectiveness of wastewater and environmental surveillance

**DOI:** 10.3389/fpubh.2026.1766749

**Published:** 2026-03-18

**Authors:** Henry H. Willis, Adeline E. Williams, Saskia Popescu, Derek Roberts, Eva Coringrato, Laura J. Faherty, Pedro Nascimento de Lima, Sana Zakaria

**Affiliations:** 1 RAND Corporation, Santa Monica, CA, United States; 2 RAND Europe, Cambridge, United Kingdom

**Keywords:** evaluation framework, infectious disease, public health, wastewater and environmental surveillance, WES, program evaluation, biosurveillance

## Abstract

Wastewater and environmental surveillance (WES) enhances infectious disease outbreak awareness by detecting pathogens before symptoms or clinical testing. As communities seek to implement, optimize, or expand WES programs, evaluation is essential to ensure effectiveness, cost efficiency, and trustworthiness. However, program evaluation guidance, including a logic model, is lacking. This conceptual analysis addresses this gap by presenting a modular, evidence-based logic model grounded in 151 WES evaluations from 2016 to 2025 across diverse contexts and program types. The model aligns with Kellogg Foundation principles and supports multiple evaluation types. It outlines inputs, activities, outputs, and outcomes leading to three ultimate goals: reducing infectious disease burden, lowering risks from catastrophic biological events, and strengthening public health system resilience. We offer recommendations on how public health agencies can use this model to guide planning and evaluation of WES implementation or expansion, ensuring stronger preparedness and response to future public health threats.

## Graphical abstract

**Figure:**
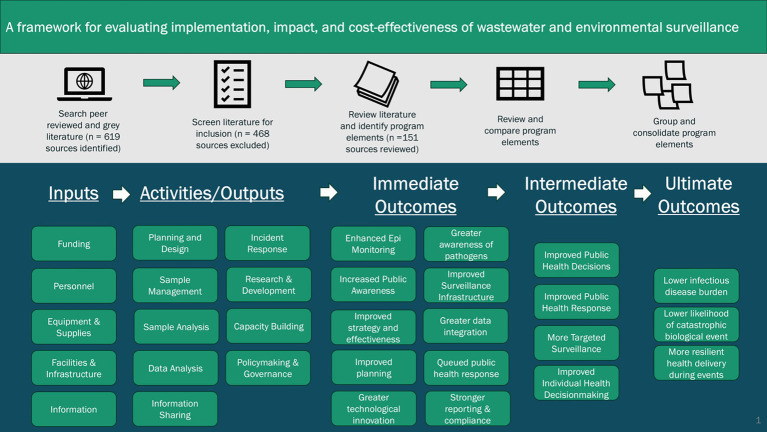

## Introduction

Wastewater and environmental surveillance (WES) is a public health tool that involves analyzing community and environmental wastewater samples for markers of disease (e.g., pathogen genomic material) or other conditions that are shed in human or animal waste. This strategy provides data on pathogen transmission dynamics and can detect outbreaks or disease trends before clinical cases are reported.

Pathogens present in feces, urine, or other bodily secretions that enter the wastewater system can be detected through WES. Examples include enteric viruses like polio or norovirus, respiratory viruses like SARS-CoV-2 and adenoviruses, enteric bacteria like *Escherichia coli* and *Vibrio cholerae*, and parasites like nematodes and flukes (1). Although many of these are transmitted through the fecal-oral route, some—such as respiratory viruses—do not readily infect people upon exposure to contaminated water. For example, we now know that SARS-CoV-2 is widely distributed in different tissue types early in infection, including in non-respiratory tissues, and that it is shed in excrement, even though it is primarily transmitted through respiratory droplets (2, 3). Indeed, WES has been more widely adopted after the COVID-19 pandemic because of its value in early detection; the method has also heightened interest in “pathogen agnostic” strategies that use non-targeted approaches that could detect novel pathogens. Previously, we found that environmental sampling surveillance technologies like WES could significantly reduce deaths during a COVID-19-like pandemic compared to syndromic surveillance alone and could also lead to a net monetary benefit (4).

As communities seek to implement, optimize, or expand WES programs, they must ensure the program includes all activities required effectively achieve the intended results and that these activities are supported with all necessary resources required. Communities must also confirm that WES results can be trusted and are generated cost effectively. Ensuring effective program design and implementation can be achieved through program evaluation (5, 6). An evaluation is “an assessment using systematic data collection and analysis of one or more programs, policies, regulations, [or] organizations intended to assess their effectiveness and efficiency (7).” However, some types of evaluations also focus on program delivery and fidelity in addition to effectiveness and efficiency. A encompassing definition can therefore be “the systematic collection of information about the activities, characteristics, and outcomes of programs to make judgements about the program, improve program effectiveness, and/or inform decisions about future programming (8).” Evaluations take varied forms to suit different contexts and needs but can be broadly understood in terms of purpose and type. With respect to purpose, evaluations may be formative, aimed at assessing and improving feasibility, appropriateness, and acceptability during program development and implementation, or summative, aimed at assessing the extent to which a program has achieved its goals (8). Within these purposes, different types of evaluation focus on a particular aspects of a program: a process evaluation assess how a program is delivered relative to its design; an outcome evaluation, assess whether intended outcomes are achieved (i.e., an outcome evaluation), attribution of causal effects by comparing to what would have happened otherwise (i.e., an impact evaluation), and benefits achieved relative to costs incurred (i.e., an economic evaluation) (9).

A foundational component of program evaluation is a complete and coherent logic model; a roadmap that explains how program resources and activities lead to intended effects (6). While previous studies have developed frameworks and tools (10) to support evaluation of public and environmental health surveillance programs, including for identifying appropriate evaluation approaches (11) and selecting data and indicators (12, 13), guidance to support WES program evaluation, including a logic model, is lacking. For example, Section 4.6 on quality assurance and continuous improvement in the WHO draft guidance on prioritization, implementation, and integration for WES consists of the following statement: “placeholder covering measurement and evaluation (14).” Because many COVID-19 WES programs were stood up during crisis, implementers did not have the convenience to perform ongoing evaluations or consider evaluability assessments. This was evident in the literature, where small-scale WES programs largely reported comparisons to clinical data without a clear evaluation framework to depend on (12).

This conceptual analysis fills this gap by proposing a dynamic evidenced-based logic model for WES that public health officials can use to develop evaluation plans consistent with guidance from the Kellogg Foundation (15, 16) to integrate planning, evaluation, and program impact. In this analysis, we performed a scoping review of scientific and grey literature, manually screened more than 600 sources for inclusion based on whether the documents were related to WES evaluations, and ultimately extracted data from 151 papers. With this information, we built a conceptual logic model that broadly outlined inputs, activities, outputs, and outcomes for diverse WES programs leading to three ultimate goals: reducing infectious disease burden, lowering risks from catastrophic biological events, and strengthening public health system resilience. Because it is modular, the logic model can be used with different types of evaluations in different contexts. Below, we present an overview of the evaluation literature upon which the logic model is based; the structure, context, and assumptions of the logic model; and recommendations on how public health officials could use the logic model in evaluating and planning adoption or expansion of WES to improve public health response to infectious disease outbreaks.

## Methodology

### Search strategy and selection criteria

The search strategy was developed by a Research Librarian and the Authors, based on example citations, keywords, concepts, and terminology shared during team discussion. The search syntax combined Boolean logic with key terms and phrases intended to facilitate relevant results for screening articles, chapters, government publications, and reports discussing the evaluation of wastewater surveillance programs.

Database and grey literature searches were completed by the Research Librarian in December 2024 and January 2025. Search strategies were peer-reviewed by another Librarian prior to execution. Scholarly literature was retrieved from PubMed (NLM) and Web of Science (Clarivate). To identify grey literature on topic, the Librarian performed targeted, iterative handsearching of the following sources: Advanced Google, bioRXiv (CSHL), and medRxiv (CSHL).

Search terms were ((program* AND (evaluat* OR assess* OR effectiv* OR “pilot” OR implement* OR optimiz* OR design)) OR “theory of change” OR “logic model” OR “cost benefit analysis” OR “logic tree”) AND ((wastewater OR “waste water” OR environmental OR travel*) AND (surveillance OR biosurveillance OR “bio surveillance” OR genet* OR genom* OR diseas* OR “early detect*” OR “early warn*” OR epidemiol*)). In Advanced Google, bioRXiv, and medRXiV multiple searches were performed using various combinations of the above noted terms and limiters.

Additionally, the following related agency websites were manually searched:

- Africa Centres for Disease Control and Prevention (Africa CDC)
- National Academies of Sciences, Engineering, and Medicine (NASEM)
- United States Centers for Disease Control and Prevention (CDC)
- World Health Organization (WHO)

Advanced Google Search was utilized to identify other relevant articles, reports, conference proceedings, policy documents, and non-commercial publications from web domains .edu, .eu, .gov, and .org.

Inclusion criteria comprised of terms found in the title, abstract, or summary indicating assessment, evaluation, and/or implementation of wastewater surveillance, biosurveillance, and/or environmental epidemiology programs. Relevant literature was found in Immunology, Pathology, and Public and Global Health research collections from preprint servers. Additional inclusion criteria: English language and published after January 1, 2014.

All database results (as .RIS files) were created or imported and compiled in an EndNote library by the Research Librarian for reference management, duplicate removal, and data management. Duplicate citations were first removed using EndNote’s “Find Duplicates” feature, followed by a manual review by the Librarian. Duplicates were identified based on matching bibliographic information, including title, author, publication year, or DOI.

### Logic model development

For each source captured in the literature review, we manually extracted relevant information into a spreadsheet to develop the logic model, including: (1) study question or objective, (2) program goal, (3) diseases/substances/markers covered, (4) financial program inputs, (5) human program inputs, (6) physical program inputs, (7) information program inputs, (8) partnership/community support program inputs, (9) regulatory program inputs, (10) program activities, (11) program outputs, (12) short-term outcomes, (13) intermediate outcomes, (14) long-term outcomes that were explicit or inferred, (15) program assumptions, (16) overall effectiveness or other conclusions, (17) challenges/issues identified, (18) regions covered, (19) country covered, (20) whether the country was an LMIC, (21) whether the paper described a technology (e.g., wastewater metagenomic sequencing) or system (WES program level) evaluation, (22) whether the paper described a modeling effort or was an empirical (i.e., observational) evaluation, (23) the application of the program (e.g., infectious disease, antimicrobial resistance (AMR), illicit drug use, etc.), (24) whether the paper was COVID-19 related, (25) study time frame, and (26) type of evaluation.

Based on this information, the author team developed a logic model that broadly captured all the components captured in the literature as well as based on prior knowledge and work. Additionally, they shared the logic model with biosurveillance implementers and experts to elicit feedback and ensure the logic model was all encompassing, as mentioned in the Acknowledgements section.

## Results

### Literature supporting WES evaluations

**Figure 1:**
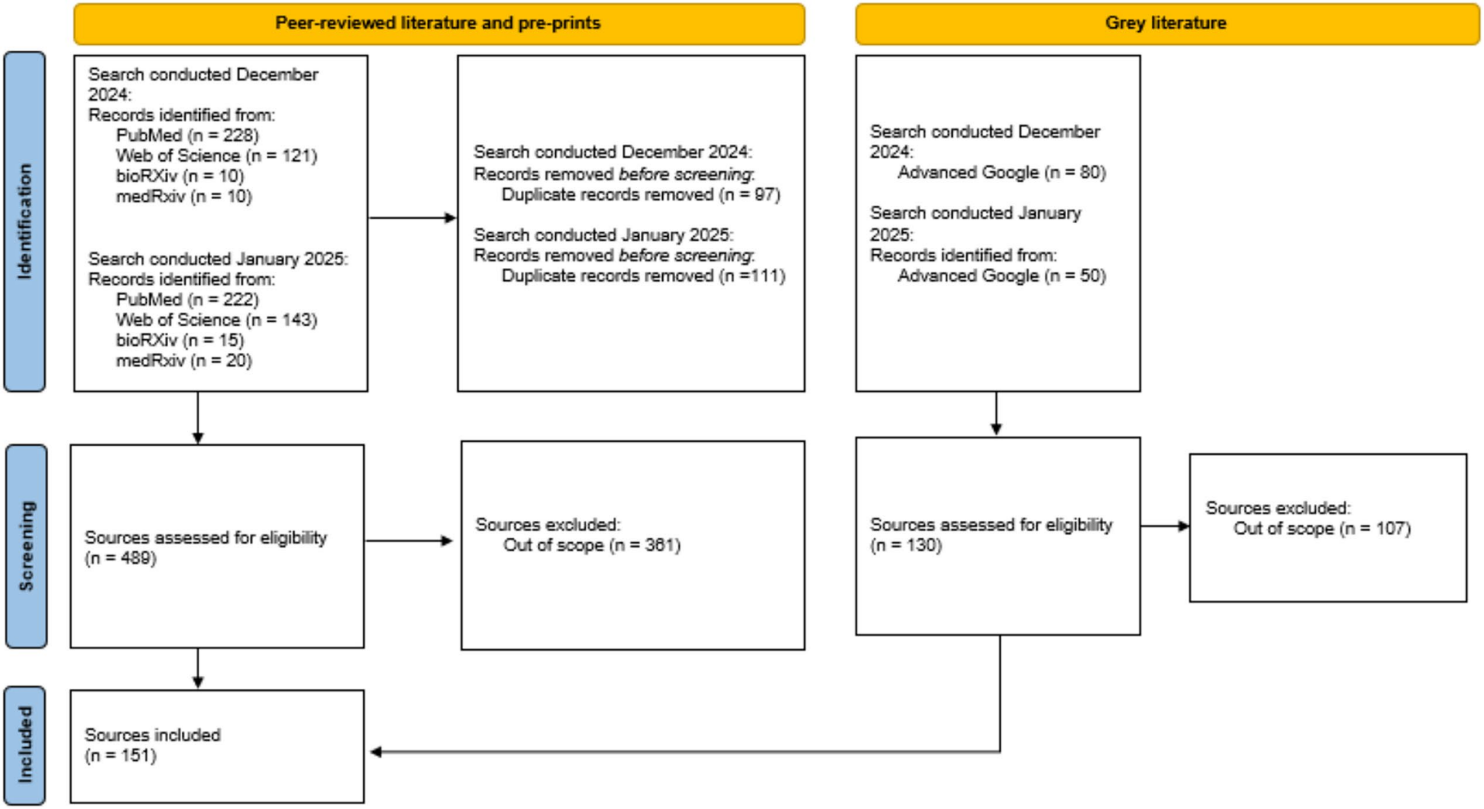
Flow diagram of literature identification, screening, and inclusion.

To better assess existing WES systems, evaluations, and applications, we performed a literature review that examined over 600 sources identified between 2016 and 2025. Of these, we narrowed the focus to 151 relevant sources—128 WES program evaluations and 23 WES guidance documents—that tested procedures or methods, established best practices, or considered key contributors to successful outcomes, results, and cost effectiveness (Supplementary Table 1). The publications were identified in PubMed, Web of Science, bioRXiv, medRXiv, and an advanced Google search and included peer-reviewed journals and reports by governmental, non-governmental, and private research organizations (Figure 1). They covered programs related to sewered, private facilities, and non-sewered communities in diverse geographies across the world aimed at detecting different types of markers (e.g., pathogens, antimicrobial drug resistance (AMR), drug use, behaviors, etc.) (Table 1). Although most literature focused on infectious disease agents, we also included WES program evaluation manuscripts related to non-infectious agents, such as illicit drug use or environmental hazards (Table 1). Because WES programs can include multiple targets, we broadly considered both infectious and non-infectious materials in our review, even though the logic model is ultimately geared to infectious disease threats.

**Table 1:** Summary of literature review findings.

Category Descriptor	Type	# of studies identified. () indicate grey literature
Application	Infectious disease	116 (21)
	SARS-CoV-2	102 (16)
	Other (e.g., mpox, influenza, polio, etc.)	31 (6)
	Antimicrobial drug resistance	10 (4)
	Illicit drug use	5 (2)
	Population behaviors	3 (2)
	Environmental hazards	2 (3)
Low- or middle-income country	Yes	20 (6)
	No	99 (13)
Type of evaluation	Formative	48 (5)
	Process	12 (7)
	Outcome	33 (3)
	Impact	6 (0)
	Economic	7 (0)
Empirical or modeling	Empirical	78 (4)
	Modeling	23 (0)
System or technology	System	104 (14)
	Technology	13 (1)

Numbers in this table do not sum to the total number of studies for two reasons: if a study fell into more than 1 category, it was counted for each category, and not every study could be categorized in each of these dimensions.

Based upon these findings, we identified major themes and trends about WES systems, outcomes, and evaluations. First, an overwhelming majority of the studies were done within the last 5 years [*n* = 126 (of 128)] with an emphasis on SARS-CoV-2 (COVID-19) surveillance [*n* = 102 (of 128)]. Evaluations primarily focused on the feasibility and efficacy of WES to monitor disease in specific populations and communities, such as university campuses (17–19), prisons (20–24), or long-term care facilities (25). For program evaluations, authors tended to measure the association between wastewater detection levels (e.g., SARS-CoV-2 RNA) and clinical cases (daily, weekly, and clinical) (26–32), although there was one study that modeled the economic efficiency of wastewater versus clinical surveillance (33). When discussing the value of WES, the sources considered how WES data could be integrated into decision-making and public health resourcing (34–38), and how WES is a complementary resource to traditional public health efforts (36, 39–42).

**Figure 2:**
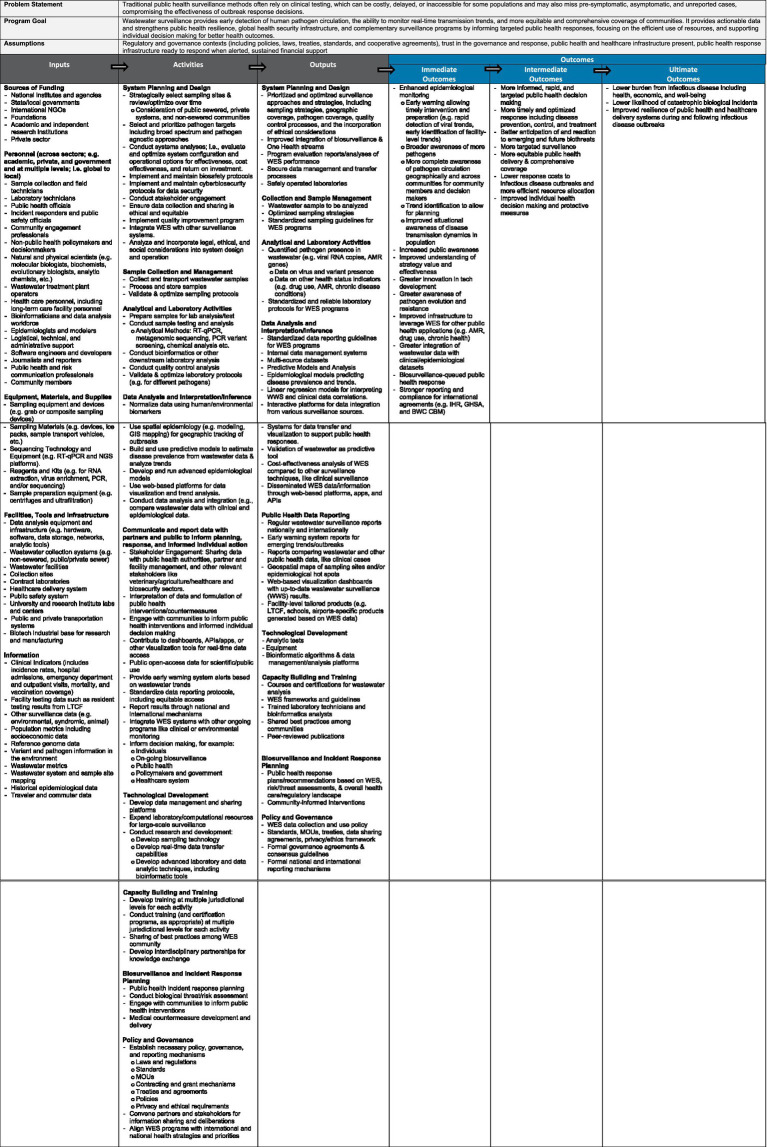
Logic model for evaluating the implementation, impact, and cost effectiveness of wastewater and environmental surveillance (WES).

We identified key themes across the literature that informed the logic model, specifically on program goals, resources, activities, outputs, and short-term/intermediate/long-term outcomes. We organized information extracted from the literature using the Kellogg logic model framework to describe the goal of the program and context within which it operates, the outcomes that support the program goal along with the activities required to achieve them, the results those activities produce, and the inputs required. The resulting logic model is provided in Figure 2 and described in the following section along with citations that provide examples of the elements identified. The logic model offers a framework to guide WES program design, evaluation, and implementation, helping communities refine goals, assess resources, and share best practices through tools like surveys and toolkits. It can also support global organizations in developing standards and guiding principles that strengthen public health resilience and advance global health security.

### Logic model structure and components

A logic model connects inputs, activities, and outputs (Figure 2, grey boxes) in a systematic way to produce a chain of outcomes (Figure 2, blue boxes)—immediate (short-term results), intermediate (behavioral or process changes), and ultimate (long-term impacts)—illustrating how resources and actions lead to desired goals. We narrowed the focus of this logic model to targeting infectious disease threats but did consider non-infectious disease literature when building the model as we were cognizant that WES programs can have multiple targets.

In this context, the overall goal of WES programs targeted to infectious disease threats is to provide “early detection of human pathogen circulation, the ability to monitor real-time transmission trends, and more equitable and comprehensive coverage of communities. It provides actionable data and strengthens public health resilience, global health security infrastructure, and complementary surveillance programs by informing targeted public health responses, focusing on the efficient use of resources, and supporting individual decision making for better health outcomes” (Figure 2, program goal). The logic model recognizes that the program operates within existing contexts of regulatory and public health/healthcare policies and programs, environments of stakeholder trust in governance and response, constraints on financial support, and public health response and infrastructure available to support response when alerted (Figure 2, assumptions). We also described the challenges of traditional syndromic surveillance that WES programs address (Figure 2, problem statement).

Given these goals, we summarized the ultimate outcomes of WES programs as (1) a lower burden from infectious disease, including health, economic, and well-being, (2) improved resilience of public health and healthcare delivery systems during and following infectious disease outbreaks, and (3) lower likelihood of catastrophic biological incidents. Routine WES contributes to lowering the burden of infectious diseases because it enables the detection of unexpected health threats prior to their evolution into epidemics, is cost effective compared to other forms of health surveillance and better protects medically vulnerable populations. The burden of disease extends beyond health and economic measures to include other wellbeing measures such as effects on educational outcomes, mental health, and reduced disruptions of daily activities. WES leads to improved resiliency by enabling more timely disease control measures, reduced rates of hospitalization, and more rapid diagnosis and treatments that can allow for more efficient use of resources. Finally, WES lowers the likelihood of catastrophic biological incidents by providing early warning and tracking of disease spread in a highly efficient manner; for example, through airport sentinel sites.

With the program goals and ultimate outcomes in mind, the logic model first starts with resources that are necessary inputs to translate plans into results (Figure 2, inputs).

#### Inputs

Inputs refer to all resources that are required to establish and maintain the WES programs. Inputs gathered from the literature were grouped into several broad categories: (1) sources of funding, (2) personnel, (3) equipment, materials, and supplies, (4) facilities, tools, and infrastructure, and (5) information. Some essential inputs, such as wastewater sample collection devices, were consistently cited in the WES program literature, but the exact type varied based on program setting. In other cases, some inputs were only necessary for particular programs, such as sequencing equipment. To produce a fully encompassing logic model, we included all inputs gathered from the literature at a high level and gave examples, keeping in mind that not all inputs will be necessary for every program.

Program funding sources ranged from international to city levels and consisted of public, private, academic, and non-profit entities. Globally, national-level government organizations were the most common funders of WES programs (26, 38). Programs frequently partnered with academic institutions for both funding and testing capabilities (31, 43), and some U.S. states (44) and counties (45) funded city or intrastate programs. WES programs in LMICs or requiring regional coordination were often partially funded by international NGOs, private foundations (40), or regional entities (30).

A wide variety of personnel are required for effective WES programs. Public health officials, local officials, and natural and physical scientists are central to the establishment of programs (46). Wastewater treatment plant operators are necessary in settings with centralized sewage systems, while field sample collection technicians are necessary in settings with less standardized sewer systems. (34) Laboratory technicians process and test samples—generating data which can be analyzed and prepared by modelers, bioinformaticians, and software developers for officials and epidemiologists (44, 47). At all program stages, community involvement is critical—an informed and invested community is more willing to tolerate inconveniences, and can ensure program continuity (48).

The bulk of WES program equipment, materials, and supplies are used for sample collection, transport, processing, and testing. Wastewater composite and automatic samplers are the gold-standard, but simple grab sample supplies are often more suitable for rural settings or urban settings with less centralized sewer systems (43, 49). Sample transport vehicles and freezer storage are necessary to preserve sample integrity after collection, while laboratory reagents, kits, and equipment are used for sample cleaning, purifying, and testing (50). Thermocyclers are used for standard PCR testing (50) and next generation sequencing machines for metagenomic analyses (51).

Facilities, tools, and infrastructure inputs span from sample collection sites to data analysis tools. Wastewater treatment facilities provide sewage system and collection site access (26). Transportation systems enable sample delivery to lab facilities for sample testing and data analysis using a variety of hardware and software tools (52). These labs are generally housed in universities (53), public health departments (54), or private, contracted companies (55). Integration with established healthcare and public health safety systems improves communication and informs healthcare delivery (35).

An extensive range of information inputs are utilized in well-designed WES programs. Epidemiological data inform all aspects of WES programs and are essential for comparison and validation (26). Data on sewer system geography and sewage dynamics, including flow rate, temperature, and rainfall dilution, are critical for program strategy and to normalize pathogen material concentrations (50, 56). The use of previously validated WES protocols for sampling, testing, and analysis increases interoperability and standardization of results (18). Census and population mobility data enable the estimation of disease incidence and can explain trends in wastewater concentrations (57). Genetic data including reference genomes and variant mutations are especially important for metagenomic sequencing (51).

#### Activities and outputs

Activities are deliberate actions, interventions and processes conducted with inputs to achieve program objectives (16, 46). Outputs are tangible products or services generated through these activities (16, 46). Our review of WES program evaluations identified a range of activities and outputs. While core components, including sampling, analysis and reporting, were common across programs, others varied based on the program’s context, aims, resources and maturity. To reflect the breadth described in the literature, we grouped activities and outputs into 11 categories, discussed below.

WES system planning and design begins early in a program’s lifecycle. It includes the strategic selection of sampling sites, accounting for sewered, private and non-sewered systems (58). System designers and planners select and prioritize pathogen targets, considering how WES systems integrate with or complement other surveillance systems and can improve integration of biosurveillance and One Health streams (59, 60). Incorporating legal, ethical and social considerations into system design and operation, as well as engaging stakeholders in system planning and operation, promotes ethical and equitable systems (61, 62). Planning also includes developing, implementing and maintaining biosafety and cybersecurity protocols, contributing to secure data management processes and safely operated systems (58). Quality improvement programs support continuous optimization, evidenced by performance evaluation reports.

WES programs collect, transport, process and store wastewater samples. Samples are then prepared, tested and analyzed, including downstream and quality control analyses. As part of quality improvement programs and system coordination efforts, sampling and analysis protocols may be developed, validated, optimized and standardized (63, 64). These activities generate wastewater samples, data on virus and variant presence, antimicrobial resistance (27, 51, 63, 65, 66), chronic conditions (67) and drug use (68, 69).

WES programs typically conduct data analysis, including normalization, spatial assessment and predictive and epidemiological modeling, to interpret and infer information generated from complex sources. A small number of sources described WES programs conducting biological threat (70) and risk analyses (71). Analytical activities may examine WES data in isolation or integrated with clinical, environmental or other surveillance data (61). Analytical activities can generate tools for data management, transfer and communication (72). WES program data may be disseminated through public and restricted access reports, dashboards, models, datasets, visualizations and interactive platforms, supported by standardized data reporting guidelines.

WES programs may communicate and report to stakeholders and the public to support planning, response and informed individual action. This may include engaging stakeholders and communities, interpretating data, contributing to real-time and open access data, providing early warning alerts, standardizing protocols, integrating WES with other monitoring systems and reporting results through national and international mechanisms (58).

WES programs generate various types of public health data reporting depending on the aims and context of the program. These reports may include data on local, national and international surveillance, emerging trends and outbreaks and comparisons with other surveillance data. Other forms of reporting include geospatial maps (49, 72, 73), web-based visualization dashboards and context-specific products including those tailored to schools (74), airports (75) and long-term care facilities (25, 76). Additionally, biosurveillance and incident response activities including risk assessments (71), response planning, medical countermeasure development and integration with other surveillance systems, generate response plans and community-informed interventions (58).

Over time, WES programs develop and refine technologies for sampling, analysis, data management and sharing. Programs may expand their laboratory and computational resources to conduct large-scale surveillance and may conduct research and development, producing new tests, equipment, bioinformatic algorithms and data management and analysis platforms.

WES programs build capacity internally and externally by developing and conducting training at multiple jurisdictional levels, developing interdisciplinary partnerships for knowledge exchange and sharing best practices within the WES community (64, 77). These activities generate training courses and certifications for wastewater analysis, frameworks and guidelines, trained staff including technicians and analysts, peer-reviewed publications and, over time, establish best practices (14).

WES programs also contribute to and are reliant on underlying policy, governance and reporting mechanisms including laws, regulations, standards, MOUs, contracting and grant mechanisms, treaties and agreements, policies and privacy and ethical requirements (44, 78). This involves convening partners and stakeholders for information sharing and deliberation, aligning WES programs with international and national health strategies and priorities.

#### Immediate outcomes

WES program inputs, activities, and outputs lead directly to outcomes. Within our review, we structured outcomes based upon temporal shifts in behavior, knowledge, and function (16). As outcomes can be measured in various timeframes, the first category focuses on those most immediate outcomes as a result of WES programs, whether that be from ground-up design and implementation to expansion of existing systems. Immediate outcomes are those that directly impact knowledge and skills but also attitudes that can later translate to behavioral changes and decision-making. Most evaluations of WES programs were formative (e.g., WES in Welsh prisons (22)) or assessed implementation (e.g., a Cypress national WES program (50),) which points to an underlying focus within emergent public health events, when awareness and rapid response are critical.

Within the logic model, these short-term outcomes included enhanced epidemiological monitoring. Improved disease surveillance has significant implications, including the capacity for rapid detection and identification of pathogens (24), which provides more complete awareness of those pathogens in circulation, but also pathogen evolution and potential resistance to antimicrobials (51). Furthermore, enhanced biosurveillance enables communities to identify epidemiological trends which can then inform immediate response but also strengthen long-term strategy and infrastructure that can broaden potential public health applications. Much of the literature reiterated improved monitoring of pathogens, like SARS-CoV-2, that allowed for improved understanding of transmission with various environments, such as schools (79) and prisons (23). With this enhanced monitoring and response capacity, there is an improved understanding of the value and effectiveness of WES, which often translates to increased public awareness. For example, in Norway, this translated to a stronger understanding of WES utility and monitoring of SARS-CoV-2 within the community but also emerging variants (26). WES using sequencing technology is especially promising for the tracking of pathogen evolution and variant incidence (27, 45).

The spurring of technological innovation and application and integration of WES data with clinical and epidemiological datasets also presents as a more immediate outcome of these programs. The strengthening of epidemiological monitoring and integration with such datasets not only translates to more rapid public health response and awareness (21, 80) but also has the capacity to improve reporting and compliance with international agreements such as the International Health Regulations (IHR) and Biological and Toxins Weapon Convention (BWC) (61). While awareness, increased capabilities, and response were the primary focus points of WES programs and evaluation of the systems, a byproduct we observed but was not heavily highlighted in the literature, was stronger international reporting and norms to health preparedness. Stronger reporting on national and regional scales was predominantly highlighted from larger studies, such as those by governments or multi-national organizations (81). Immediate outcomes identified within the literature were primarily focused on increasing awareness for the feasibility and effectiveness of WES as a public health and outbreak tool, especially during the COVID-19 pandemic, which improved overall understanding of epidemiological trends while allowing for more prompt identification of various pathogens.

#### Intermediate outcomes

Intermediate outcomes reflect behavioral changes that occur when newly gained information manifests into actionable change. While immediate outcomes occur more rapidly and are often short-term, intermediate outcomes are likely to occur more slowly and build on those immediate outcomes. The logic model underscores these intermediate changes through behavioral shifts that build on increased public awareness and identification of trends, to develop more timely and strategic health response because of the WES programs and findings. Intermediate outcomes may take longer, often several years, but are broader and reflect skills development and behavior changes, like improved decision-making for individuals regarding personal risk and interventions. The knowledge and attitude changes that are immediate outcomes, directly support larger skills and functionality shifts that allow for more widescale, long-lasting change. We identified several intermediate outcomes within the literature, including a commonality of more informed, rapid, and targeted decision-making, both for public health, but also individuals making personal health decisions. WES programs can improve public health decision making and resource management (e.g., tests) (38). A one-year review of the “COVIDPoops19” global dashboard found that publicly accessible WES data can guide both individual risk decisions and broader public health responses (82). Much of the literature underscored intermediate outcomes during outbreaks through more targeted and rapid response measures. For example, a study in Spain found that SARS-CoV-2 WES informed government restrictions, helped hospitals prepare for case surges, and guided industry planning during the COVID-19 pandemic (83).

The most supported intermediate outcome within the literature was the development **more** targeted surveillance and timely and optimized response, including disease prevention, control, and treatment. The importance of WES programs and improved programmatic public health responses was particularly highlighted across LMICs, including efforts to increase response to polio. Efforts included targeted response efforts such as supplemental immunization campaigns (84), interrupted transmission of circulating vaccine-derived poliovirus type 2 (cVDPV2) in the northern region of Ghana (34), and WES program-enable interruption of poliovirus transmission in South East Asia and inform improved understanding of which immunization activities are likely to be most effective within a given population (77). Furthermore, WES programs helped inform community stakeholders on various issues, including nutritional status of the population, allowing them to design targeted strategies (67) but also inform policy action and investment of resources; like that necessary to understand and address AMR globally (64). A consistent trend in the literature was the role of WES programs in enabling the design and refinement of more targeted deployment of public health resources and efforts and community-focused outreach and vaccination campaigns (36). These efforts led to increased rates and location-specific focus for vaccination in hot spots, prompted by multiple positive detections (85).

In the literature, the role of WES programs in promoting more equitable public health delivery and comprehensive coverage is frequently discussed as an aspiration (86, 87), with fewer studies assessing the realization of this outcome. The literature suggests that WES programs, through enhanced epidemiological monitoring across pathogens, geographies and communities, can extend surveillance to populations poorly covered by other approaches. Modelling studies provide evidence for the potential of WES programs to support more equitable public health delivery. For example, a geospatial analysis examining the differences in sewered and un-sewered populations found WES can improve health equity by better capturing vulnerable populations compared to clinical data (88). Studies that considered longer-term outcomes provided evidence of how WES promotes more equitable public health delivery. A study utilizing public schools as community sentinel surveillance sites for SARS-CoV-2 found that WES programs can support community-tailored interventions, such as ensuring materials are translated into languages spoken locally, working with field staff to ensure culturally competent outreach, and providing community-specific testing and vaccine clinics (45).

Evidence from the literature suggests that WES programs can contribute to lower response costs (89) and more efficient resource allocation (38), although this was the least frequently mentioned intermediate outcome, likely reflecting the limited number of economic evaluations in the field. Where examined, findings indicated that by providing early warning of pathogen presence and broader geographic and demographic coverage, WES can enable targeted, timely interventions that reduce the scale and duration of outbreaks (84). This was particularly noted in LMIC contexts, where WES allowed for the collection of non-identifiable health information from communities without access to clinical testing (84, 90). Broader surveillance coverage provided a more complete understanding of pathogen circulation at lower cost than individual diagnostic testing, informing more strategic allocation of limited public health resources (90).

#### Ultimate outcomes

Ultimate outcomes are long-term, semi-permanent, and broadly impactful outcomes that frequently manifest alongside changes to deeply rooted systems, reflecting change outside the scope of a single WES program. Ultimate outcomes were infrequently mentioned in the literature, reflecting a paucity of impact evaluations, and some required a level of inference. The most frequently mentioned ultimate outcome was a decreased burden from infectious disease, arising from early detection of health threats (37), cost-efficiency compared to other forms of health surveillance (28), and broad population coverage, contributing to a reduction of health inequalities and better protection of medically vulnerable populations (37, 91). A less frequently mentioned, but often implied, outcome was improved resilience of public health and healthcare delivery systems during and following infectious disease outbreaks, arising from the synergistic effects of more informed, rapid and targeted response measures (26), improved diagnosis and healthcare delivery through clinical data integration (63, 65), and more optimized resource allocation (92). The literature also suggested that implementation of WES programs can contribute to a lower likelihood of catastrophic biological incidents. As part of interconnected systems for health and environmental surveillance, WES programs contribute to early warning and disease tracking (93, 94), pathogen variant identification and characterization (30), and, in some cases, disease eradication (34).

#### Contexts and stakeholders

The range of WES programs and corresponding evaluations led to considerable variety within the logic model, underscoring the inter-relatedness between some components, for example, the co-occurrence of the metagenomic sequencing activity and the increased awareness of pathogen evolution outcome. Utilization of the logic model will depend upon several indicators, such as location (HIC vs. LMIC), a sewered or non-sewered system, use within certain communities or settings (e.g., universities, prisons, nursing homes, regions, etc.), and if the focus of the WES system is for non-infectious disease applications, such as illicit drug use, population health behaviors, or environmental hazards. Many WES evaluations involved globally diverse programs focused on COVID-19 biosurveillance, which included outputs and outcomes focused on public health data reporting, timely and improved public health delivery, and capacity building in response to the pandemic. Whereas those with applications in community behaviors, such as illicit drug use, involved system evaluations in the United States and China, with short-term outcomes focused on pivoting WES systems for such applications (68, 69). Many of the evaluations were done to test efficacy and robustness of programs recently implemented or operationalized for specific purposes, such as COVID-19. As such, the logic model is particularly geared towards those seeking to establish new or expand existing WES programs in which there is a near-term need for increased awareness and knowledge, enhanced biosurveillance utilization and integration, and public health response. While various components of the logic model, such as the specific inputs and outputs, may change based upon stakeholder needs and resources, the logic model outcomes were relatively consistent across the literature.

### Future applications of the logic model

The logic model presented in this manuscript provides a framework for both WES program implementation and evaluation. It is built on the evidence documented as communities around the world sought to develop and use innovative methods of surveillance to improve public health awareness and response. As this progress continues, the logic model can be used in several ways to continue advancements in global health security.

First, the logic model provides a mechanism for helping communities sharpen the focus of their WES program goals and intended outcomes and ensure implementation includes the resources and supporting activities required to achieve them. To this end, we are developing a survey based on the logic model that communities can self-administer. The survey results will summarize the focus and scope of a program design and can be used to assess the completeness and adequacy of each. As more communities use and share results from such a survey, results can help to build awareness about the extent of and best practices for WES implementation.

Second, organizations such as the World Health Organization and the European Union’s Health Emergency Response Agency are developing guides and standards for WES program implementation. As these efforts continue, their results can be assessed within the context of this logic model to build consensus around the theory of change for WES. Awareness of WES program implementation gained through the aforementioned survey can also over time be incorporated into this guidance.

As awareness of WES implementation and best practices expand, the logic model can be translated into tools to support program design and evaluation. For example, checklists of possible program outcomes can help programs ensure they are setting themselves up to reach the maximum potential value. Additionally, checklists of supporting resources and activities can help planners confirm and document any gaps that need to be filled to ensure a successful WES program. To this end, we aim to develop a toolkit that stakeholders can engage with to facilitate program planning, decision making, and evaluation. We anticipate that this tool could also be incorporated into guiding principles for developing new programs, coordinating with existing ones, and monitoring them throughout their life cycle.

As we work to develop and use this survey and toolkit, we invite others to engage with this proposed WES logic model sharing ways that it can be enhanced and how its use helps to provide actionable data to strengthen public health resilience and global health security.

### Limitations

Although our literature review was scoping and included a broad set of terms, we did not perform a systematic review. We may have missed manuscripts using key words that were not included in our search, such as “technology” or “intervention.” We also only included manuscripts from 2014 to 2025, so we could have missed relevant evaluation manuscripts that were published prior to that time frame.
