# Supplementary material for: A framework for evaluating implementation, impact, and cost-effectiveness of wastewater and environmental surveillance
Source: Front Public Health. 2026 Mar 18;14:1766749. doi: 10.3389/fpubh.2026.1766749 (PMC13038939; doi:10.3389/fpubh.2026.1766749)
Supplement: Supplementary file 1 [file Table_1.docx]

**Supplementary Table 1:** Sources included in the literature review of evaluations of wastewater surveillance interventions

| Type of Evaluation | Year | Author | Title | Reference Type |
| --- | --- | --- | --- | --- |
| Economic | 2023 | B. K. Yoo, R. Iwamoto, U. Chung, T. Sasaki and M. Kitajima | Economic Evaluation of Wastewater Surveillance Combined with Clinical COVID-19 Screening Tests, Japan | Journal Article |
| Economic | 2023 | N. Thampi, E. Mercier, B. Paes, J. O. Edwards, B. Rodgers-Gray and R. Delatolla | Perspective: the potential of wastewater-based surveillance as an economically feasible game changer in reducing the global burden of pediatric respiratory syncytial virus infection | Journal Article |
| Economic | 2023 | Xindi C. Hu, Aparna Keshaviah and Emily B. Harrison | The Costs of Wastewater Monitoring in Low- and Middle-Income Countries | Report |
| Formative | 2025 | G. Johnson, A. Espàrza, E. Stevenson, L. Stadler, K. Ensor, S. Williams, K. Sheth, C. Johnson and L. Hopkins | Schools and Wastewater Surveillance: Practical Implications for an Emerging Technology to Impact Child Health | Journal Article |
| Formative | 2025 | Nicole Acosta, Alex Buchner Beaudet, Paul Westlund, Jia Hu, Navid Sedaghat, Puja Pradhan, Lawrence Man, Jordan Hollman, María A. Bautista, Barbara J. Waddell, Janine McCalder, Matthew Penney, Jianwei Chen, Jon Meddings, Gopal Achari, M. Cathryn Ryan, Dany Breton, Elizabeth A. Wood, Jason L. Cabaj, Rhonda C. Clark, Kevin Frankowski, Casey R. J. Hubert and Michael D. Parkins | Assessing the performance and suitability of wastewater based-surveillance for SARS-CoV-2 RNA in public schools | Journal Article |
| Formative | 2024 | Anika John, David Dreifuss, Seju Kang, Anna Bratus-Neuenschwander, Natalia Zajac, Ivan Topolsky, Arthur Dondi, Catharine Aquino, Timothy R. Julian and Niko Beerenwinkel | Assessing different next-generation sequencing technologies for wastewater-based epidemiology | Journal Article |
| Formative | 2024 | Anna Loenenbach, Ann-Sophie Lehfeld, Peter Puetz, Barbara Biere, Susan Abunijela, Silke Buda, Michaela Diercke, Ralf Dürrwald, Timo Greiner, Walter Haas, Maria Helmrich, Kerstin Prahm, Jakob Schumacher, Marianne Wedde and Udo Buchholz | Participatory, Virologic, and Wastewater Surveillance Data to Assess Underestimation of COVID-19 Incidence, Germany, 2020–2024 | Journal Article |
| Formative | 2024 | B. R. Haskell, H. A. Dhiyebi, N. Srikanthan, L. M. Bragg, W. J. Parker, J. P. Giesy and M. R. Servos | Implementing an adaptive, two-tiered SARS-CoV-2 wastewater surveillance program on a university campus using passive sampling | Journal Article |
| Formative | 2024 | Betsy Foxman, Elizabeth Salzman, Chelsie Gesierich, Sarah Gardner, Michelle Ammerman, Marisa Eisenberg and Krista Wigginton | Wastewater surveillance of antibiotic resistant bacteria for public health action: Potential and Challenges | Journal Article |
| Formative | 2024 | G. Jones, A. Nelson, D. R. Chadwick, S. Cobley, D. L. Jones, S. Perrett, W. B. Perry, A. J. Weightman, R. C. Williams and D. R. Thomas | Evaluation of wastewater surveillance for SARS-CoV-2 in a prison population: a mixed-methods approach | Journal Article |
| Formative | 2024 | Gillian Maree, Fiona Els, Yashena Naidoo, Laven Naidoo, Phemelo Mahamuza, Mokgaetji Macheke, Nkosenhle Ndlovu, Said Rachida, Chinwe Iwu-Jaja, Setshaba Taukobong, Sibonginkosi Maposa, Kathleen O’Reilly, Mukhlid Yousif and Kerrigan McCarthy | Wastewater surveillance overcomes socio-economic limitations of laboratory-based surveillance when monitoring disease transmission: the South African experience during the COVID-19 pandemic | Journal Article |
| Formative | 2024 | HJ Smith, RT Agans and WJ Kowallis | Ethical Considerations for Wastewater Surveillance in the United States Department of Defense | Journal Article |
| Formative | 2024 | Kirsi-Maarit Lehto, Annika Länsivaara, Rafiqul Hyder, Oskari Luomala, Anssi Lipponen, Anna-Maria Hokajärvi, Annamari Heikinheimo, Tarja Pitkänen and Sami Oikarinen | Wastewater-based surveillance is an efficient monitoring tool for tracking influenza A in the community | Journal Article |
| Formative | 2024 | L. Li, L. Haak, M. Carine and K. R. Pagilla | Temporal assessment of SARS-CoV-2 detection in wastewater and its epidemiological implications in COVID-19 case dynamics | Journal Article |
| Formative | 2024 | M. J. Osborn, S. Champeau, C. Meyer, M. Hayden, L. Landini, S. Stark, S. Preekett, S. Vetter, Z. Zirnhelt, S. Meyer, D. Huff, T. W. Schacker and C. R. Doss | Wastewater Surveillance of SARS-CoV-2 in Minnesota | Journal Article |
| Formative | 2024 | Maria L. Daza-Torres, J. Cricelio Montesinos-López, César Herrera, Yury E. García, Colleen C. Naughton, Heather N. Bischel and Miriam Nuño | Optimizing spatial distribution of wastewater-based epidemiology to advance health equity | Journal Article |
| Formative | 2024 | Martin Tay, Benjamin Lee, Muhammad Hafiz Ismail, Jerald Yam, Dzulkhairul Maliki, Karina Yew-Hoong Gin, Sae-Rom Chae, Zheng Jie Marc Ho, Yee Leong Teoh, Lee Ching Ng and Judith Chui Ching Wong | Monitoring multi-pathogens and SARS-CoV-2 variants in aircraft and airport wastewater | Journal Article |
| Formative | 2024 | P. Radvák, D. Rusňáková, T. Sedláčková, M. Böhmer, A. Kaliňáková, B. Kotvasová, T. Sládeček, J. Sitarčík, J. Martiš, J. Gašper, L. Kunštek, M. Prívara, J. Budiš, A. Krivjanská, J. Turňa and T. Szemes | Evaluation of wastewater surveillance results for SARS-CoV-2 at the national scale in the Slovak Republic | Journal Article |
| Formative | 2024 | Padmini Ramachandran, Tunc Kayikcioglu, Tamara Walsky, Kathryn Judy, Jasmine Amirzadegan, Candace Hope Bias, Bereket Tesfaldet, Maria Balkey, Dietrich EppSchmidt, Hugh Rand, James Pettengill, Sandra Tallent, Eric Brown, Tina Pfefer, Ruth Timme, Amanda Windsor, Christopher Grim and Maria Hoffmann | Harnessing methods, data analysis, and near-real-time wastewater monitoring for enhanced public health response using high throughput sequencing | Journal Article |
| Formative | 2024 | R. E. Timme, J. Woods, J. L. Jones, K. R. Calci, R. Rodriguez, C. Barnes, E. Leard, M. Craven, H. Chen, C. Boerner, C. Grim, A. M. Windsor, P. Ramachandran, T. Muruvanda, H. Rand, B. Tesfaldet, J. Amirzadegan, T. Kayikcioglu, T. Walsky, M. Allard, M. Balkey, C. H. Bias, E. Brown, K. Judy, T. Pfefer, S. M. Tallent, M. Hoffmann and J. Pettengill | SARS-CoV-2 wastewater variant surveillance: pandemic response leveraging FDA's GenomeTrakr network | Journal Article |
| Formative | 2024 | Renée Street, Angela Mathee, Tarylee Reddy, Nomfundo T. Mahlangeni, Noluxabiso Mangwana, Sizwe Nkambule, Candice Webster, Stephanie Dias, Jyoti Rajan Sharma, Pritika Ramharack, Johan Louw, Swastika Surujlal-Naicker, Natacha Berkowitz, Mongezi Mdhluli, Glenda Gray, Christo Muller and Rabia Johnson | One Year of Wastewater Surveillance in South Africa Supporting COVID-19 Clinical Findings Across Two Waves of Infection | Journal Article |
| Formative | 2024 | S. E. Philo, K. B. De León, R. T. Noble, N. A. Zhou, R. Alghafri, I. Bar-Or, A. Darling, N. D'Souza, O. Hachimi, D. Kaya, S. Kim, K. Gaardbo Kuhn, B. A. Layton, C. Mansfeldt, B. Oceguera, T. S. Radniecki, J. L. Ram, L. P. Saunders, A. Shrestha, L. B. Stadler, J. A. Steele, B. S. Stevenson, J. R. Vogel, K. Bibby, A. B. Boehm, R. U. Halden and J. Delgado Vela | Wastewater surveillance for bacterial targets: current challenges and future goals | Journal Article |
| Formative | 2024 | Simon L. Grimm, Jeff T. Kaufman, Daniel P. Rice, Charles Whittaker, William J. Bradshaw and Michael R. McLaren | Inferring the sensitivity of wastewater metagenomic sequencing for virus detection and monitoring | Journal Article |
| Formative | 2024 | W. B. Perry, M. C. Chrispim, M. R. F. Barbosa, M. de Souza Lauretto, M. T. P. Razzolini, A. C. Nardocci, O. Jones, D. L. Jones, A. Weightman, M. I. Z. Sato, C. Montagner and I. Durance | Cross-continental comparative experiences of wastewater surveillance and a vision for the 21st century | Journal Article |
| Formative | 2023 | A. L. Rainey, K. Buschang, A. O'Connor, D. Love, A. M. Wormington, R. L. Messcher, J. C. Loeb, S. E. Robinson, H. Ponder, S. Waldo, R. Williams, J. Shapiro, E. B. McAlister, M. Lauzardo, J. A. Lednicky, A. T. Maurelli, T. Sabo-Attwood and J. H. Bisesi, Jr. | Retrospective Analysis of Wastewater-Based Epidemiology of SARS-CoV-2 in Residences on a Large College Campus: Relationships between Wastewater Outcomes and COVID-19 Cases across Two Semesters with Different COVID-19 Mitigation Policies | Journal Article |
| Formative | 2023 | C. H. Wong, Z. Zhang, W. Eid, J. Plaza-Diaz, P. Kabir, S. Wan, J. J. Jia, E. Mercier, O. Thakali, L. Pisharody, N. Hegazy, S. E. Stephenson, W. Fang, T. B. Nguyen, N. T. Ramsay, R. M. McKay, R. Corchis-Scott, A. E. MacKenzie, T. E. Graber, D' Aoust PM and R. Delatolla | Rapidly developed, optimized, and applied wastewater surveillance system for real-time monitoring of low-incidence, high-impact MPOX outbreak | Journal Article |
| Formative | 2023 | Dan Han, Pamela Linares, Rochelle H. Holm, Kartik Chandran and Ted Smith | Projections of wastewater as an indicator of COVID-19 cases in corrections facilities: a modelling study | Journal Article |
| Formative | 2023 | E. Amato, S. Hyllestad, P. Heradstveit, P. Langlete, L. V. Moen, A. Rohringer, J. Pires, J. A. Baz Lomba, K. Bragstad, S. L. Feruglio, P. Aavitsland and E. H. Madslien | Evaluation of the pilot wastewater surveillance for SARS-CoV-2 in Norway, June 2022 - March 2023 | Journal Article |
| Formative | 2023 | Ettore Amato, Susanne Hyllestad, Petter Heradstveit, Petter Langlete, Line Victoria Moen, Andreas Rohringer, João Pires, Jose Antonio Baz Lomba, Karoline Bragstad, Siri Laura Feruglio, Preben Aavitsland and Elisabeth Henie Madslien | Evaluation of the Pilot Wastewater Surveillance for SARS-CoV-2 in Norway, June 2022 – March 2023 | Journal Article |
| Formative | 2023 | J. W. Keck, J. Lindner, M. Liversedge, B. Mijatovic, C. Olsson, W. Strike, A. Noble, R. Adatorwovor, P. Lacy, T. Smith and S. M. Berry | Wastewater Surveillance for SARS-CoV-2 at Long-Term Care Facilities: Mixed Methods Evaluation | Journal Article |
| Formative | 2023 | M. Marques Dos Santos, L. Caixia and S. A. Snyder | Evaluation of wastewater-based epidemiology of COVID-19 approaches in Singapore's 'closed-system' scenario: A long-term country-wide assessment | Journal Article |
| Formative | 2023 | M. Neyra, D. T. Hill, L. J. Bennett, C. N. Dunham and D. A. Larsen | Establishing a Statewide Wastewater Surveillance System in Response to the COVID-19 Pandemic: A Reliable Model for Continuous and Emerging Public Health Threats | Journal Article |
| Formative | 2023 | Marlene K. Wolfe, Alexander T. Yu, Dorothea Duong, Madhura S. Rane, Bridgette Hughes, Vikram Chan-Herur, Marisa Donnelly, Shua Chai, Bradley J. White, Duc J. Vugia and Alexandria B. Boehm | Use of Wastewater for Mpox Outbreak Surveillance in California | Journal Article |
| Formative | 2023 | Meri R. J. Varkila, Maria E. Montez-Rath, Joshua A. Salomon, Xue Yu, Geoffrey A. Block, Douglas K. Owens, Glenn M. Chertow, Julie Parsonnet and Shuchi Anand | Use of Wastewater Metrics to Track COVID-19 in the US | Journal Article |
| Formative | 2023 | R. M. Klevens, C. C. W. Young, S. W. Olesen, A. Osinski, D. Church, J. Muten, L. R. Chou, T. Segal and K. Cranston | Evaluation of wastewater surveillance for SARS-CoV-2 in Massachusetts correctional facilities, 2020-2022 | Journal Article |
| Formative | 2023 | Robert C. Morfino, Stephen M. Bart, Andrew Franklin, Benjamin H. Rome, Andrew P. Rothstein, Thomas W. S. Aichele, Siyao Lisa Li, Aaron Bivins, Ezra T. Ernst and Cindy R. Friedman | Notes from the Field: Aircraft Wastewater Surveillance for Early Detection of SARS-CoV-2 Variants — John F. Kennedy International Airport, New York City, August–September 2022 | Journal Article |
| Formative | 2023 | S. Kennedy, A. C. Spaulding and Swanss writing group * | Four Models of Wastewater-Based Surveillance for SARS-CoV-2 in Jail Settings: How Monitoring Wastewater Complements Individual Screening | Journal Article |
| Formative | 2022 | M. C. B. Otero, L. A. E. Murao, M. A. G. Limen, D. R. A. Caalim, P. L. A. Gaite, M. G. Bacus, J. T. Acaso, R. M. Miguel, K. Corazo, I. E. Knot, H. Sajonia, 2nd, F. L. de Los Reyes, 3rd, C. M. B. Jaraula, E. S. Baja and D. M. N. Del Mundo | Multifaceted Assessment of Wastewater-Based Epidemiology for SARS-CoV-2 in Selected Urban Communities in Davao City, Philippines: A Pilot Study | Journal Article |
| Formative | 2022 | Maria Virginia Prieto Riquelme, Emily Garner, Suraj Gupta, Jake Metch, Ni Zhu, Matthew F. Blair, Gustavo Arango-Argoty, Ayella Maile-Moskowitz, An-dong Li, Carl-Fredrik Flach, Diana S. Aga, Indumathi M. Nambi, D. G. Joakim Larsson, Helmut Bürgmann, Tong Zhang, Amy Pruden and Peter J. Vikesland | Demonstrating a Comprehensive Wastewater-Based Surveillance Approach That Differentiates Globally Sourced Resistomes | Journal Article |
| Formative | 2022 | Shokoofeh Nourbakhsh, Aamir Fazil, Michael Li, Chand S. Mangat, Shelley W. Peterson, Jade Daigle, Stacie Langner, Jayson Shurgold, Patrick D’Aoust, Robert Delatolla, Elizabeth Mercier, Xiaoli Pang, Bonita E. Lee, Rebecca Stuart, Shinthuja Wijayasri and David Champredon | A wastewater-based epidemic model for SARS-CoV-2 with application to three Canadian cities | Journal Article |
| Formative | 2021 | C. McCall, H. Wu, E. O'Brien and I. Xagoraraki | Assessment of enteric viruses during a hepatitis outbreak in Detroit MI using wastewater surveillance and metagenomic analysis | Journal Article |
| Formative | 2021 | M. Rojas-Bonilla, A. Coulliette-Salmond, H. Belgasmi, K. Wong, L. Sayyad, E. Vega, F. Grimoldi, M. S. Oberste and R. Rüttimann | Environmental Surveillance for Risk Assessment in the Context of a Phase 2 Clinical Trial of Type 2 Novel Oral Polio Vaccine in Panama | Journal Article |
| Formative | 2021 | Natalie Sims, Lisa Avery and Barbara Kasprzyk-Hordern | Review of wastewater monitoring applications for public health and novel aspects of environmental quality | Report |
| Formative, Economic | 2022 | A. L. Rainey, J. C. Loeb, S. E. Robinson, P. Davis, S. Liang, J. A. Lednicky, E. S. Coker, T. Sabo-Attwood, J. H. Bisesi, Jr. and A. T. Maurelli | Assessment of a mass balance equation for estimating community-level prevalence of COVID-19 using wastewater-based epidemiology in a mid-sized city | Journal Article |
| Formative, Economic | 2022 | W. L. Chen and K. Bibby | Model-Based Theoretical Evaluation of the Feasibility of Using Wastewater-Based Epidemiology to Monitor Monkeypox | Journal Article |
| Formative, Impact | 2024 | Cathal Mills, Marc Chadeau-Hyam, Paul Elliott and Christl A Donnelly | The utility of wastewater surveillance for monitoring SARS-CoV-2 prevalence | Journal Article |
| Formative, Impact | 2024 | Masahiko Haraguchi, Fayette Klaassen, Ted Cohen, Joshua A. Salomon and Nicolas A. Menzies | Statistical Relationship Between Wastewater Data and Case Notifications for COVID-19 Surveillance in the United States, 2020-2023: A Bayesian Hierarchical Model | Journal Article |
| Formative, Impact | 2022 | B. A. Layton, D. Kaya, C. Kelly, K. J. Williamson, D. Alegre, S. M. Bachhuber, P. G. Banwarth, J. W. Bethel, K. Carter, B. D. Dalziel, M. Dasenko, M. Geniza, A. George, A. M. Girard, R. Haggerty, K. A. Higley, D. M. Hynes, J. Lubchenco, K. R. McLaughlin, F. J. Nieto, A. Noakes, M. Peterson, A. D. Piemonti, J. L. Sanders, B. M. Tyler and T. S. Radniecki | Evaluation of a Wastewater-Based Epidemiological Approach to Estimate the Prevalence of SARS-CoV-2 Infections and the Detection of Viral Variants in Disparate Oregon Communities at City and Neighborhood Scales | Journal Article |
| Formative, Implementation | 2021 | Sally Gutierrez, Kathryn Kazior and Smiti Nepal | A Compendium of U.S. Wastewater Surveillance to Support COVID-19 Public Health Response | Report |
| Formative, Implementation, Outcome | 2022 | Water Environment Federation | Summary Report - Pilot Program for Onsite Testing of SARS-CoV-2 in Correctional Facility Wastewater | Report |
| Formative, Outcome | 2023 | Y. M. Brooks, B. Gryskwicz, E. Sidaway, B. Shelley, L. Coroi, M. Downing, T. Downing, S. McDonnell, D. Ostrye, K. Hoop and G. Parrish | A case study of a community-organized wastewater surveillance in a small community: correlating weekly reported COVID-19 cases with SARS-CoV-2 RNA concentrations during fall 2020 to summer 2021 in Yarmouth, ME | Journal Article |
| Formative, Outcome | 2023 | Zuzana Bohrerova, Nichole E. Brinkman, Ritu Chakravarti, Saurabh Chattopadhyay, Seth A. Faith, Jay Garland, James Herrin, Natalie Hull, Michael Jahne, Dae Wook Kang, Scott P. Keely, Jiyoung Lee, Stan Lemeshow, John Lenhart, Eva Lytmer, Devesh Malgave, Lin Miao, Angela Minard-Smith, Xiaozhen Mou, Maitreyi Nagarkar, Anda Quintero, Francesca D. R. Savona, John Senko, Joan L. Slonczewski, Rachel R. Spurbeck, Michael G. Sovic, R. Travis Travis, Linda K. Weavers and Mark Weir | Ohio Coronavirus Wastewater Monitoring Network: Implementation of Statewide Monitoring for Protecting Public Health | Journal Article |
| Formative, Outcome | 2022 | B. A. Wartell, C. Proano, L. Bakalian, D. Kaya, K. Croft, M. McCreary, N. Lichtenstein, V. Miske, P. Arcellana, J. Boyer, I. V. Benschoten, M. Anderson, A. Crabb, S. Gilson, A. Gourley, T. Wheeler, B. Trest, G. Bowman and B. V. Kjellerup | Implementing wastewater surveillance for SARS-CoV-2 on a university campus: Lessons learned | Journal Article |
| Formative, Outcome | 2022 | M. Jakariya, F. Ahmed, M. A. Islam, A. Al Marzan, M. N. Hasan, M. Hossain, T. Ahmed, A. Hossain, H. M. Reza, F. Hossen, T. Nahla, M. M. Rahman, N. M. Bahadur, M. T. Islam, M. Didar-Ul-Alam, N. Mow, H. Jahan, D. Barceló, K. Bibby and P. Bhattacharya | Wastewater-based epidemiological surveillance to monitor the prevalence of SARS-CoV-2 in developing countries with onsite sanitation facilities | Journal Article |
| Formative, Outcome | 2021 | B.M. Gawlik, S. Tavazzi, G. Mariani, H. Skejo, M. Sponar, T. Higgins, G. Medema and T. Wintgens | SARS-CoV-2 Surveillance Employing Sewage Towards a Sentinel System: Feasibility assessment of an EU approach | Report |
| Formative, Outcome | 2021 | S. Karthikeyan, A. Nguyen, D. McDonald, Y. Zong, N. Ronquillo, J. Ren, J. Zou, S. Farmer, G. Humphrey, D. Henderson, T. Javidi, K. Messer, C. Anderson, R. Schooley, N. K. Martin and R. Knight | Rapid, Large-Scale Wastewater Surveillance and Automated Reporting System Enable Early Detection of Nearly 85% of COVID-19 Cases on a University Campus | Journal Article |
| Formative, Outcome, Economic | 2025 | Guillaume St-Onge, Jessica T. Davis, Laurent Hébert-Dufresne, Antoine Allard, Alessandra Urbinati, Samuel V. Scarpino, Matteo Chinazzi and Alessandro Vespignani | Pandemic monitoring with global aircraft-based wastewater surveillance networks | Journal Article |
| Formative, Outcome, Economic | 2023 | Cathal Mills, Marc Chadeau-Hyam, Paul Elliott and Christl A. Donnelly | Strategic use of SARS-CoV-2 wastewater concentration data could enhance, but not replace, high-resolution community prevalence survey programmes | Journal Article |
| Formative, Outcome, Economic | 2021 | Rafael Sanjuán and Pilar Domingo-Calap | Reliability of Wastewater Analysis for Monitoring COVID-19 Incidence Revealed by a Long-Term Follow-Up Study | Journal Article |
| Formative, Process, Outcome | 2024 | Popi Karaolia, Annalisa Quattrocchi, Jan Richter, Panagiota Loutsiou, Iakovos C. Iakovides, Anaxagoras Violaris, Kyriakos Manoli, Costas Michael, Christina Christodoulou, Pavlos Pavlou, Marios Kyriakou, Demetrios G. Eliades, Christos Haralambous, Elisavet Constantinou, Stella G. Michael, Angeliki Larcou-Yiannakou, Georgios Nikolopoulos and Despo Fatta-Kassinos | Leveraging wastewater: Validating the national-scale SARS-CoV-2 surveillance system in Cyprus for elevated public health surveillance and enhanced epidemiological insight | Journal Article |
| Impact | 2023 | Elana M. G. Chan, Amanda Bidwell, Zongxi Li, Sebastien Tilmans and Alexandria B. Boehm | Policy impact evaluation: A potential use case for longitudinal monitoring of viruses in wastewater at small geographic scales | Journal Article |
| Implementation | 2024 | Arnoldo Armenta-Castro, Mariel Araceli Oyervides-Muñoz, Alberto Aguayo-Acosta, Sofia Liliana Lucero-Saucedo, Alejandro Robles-Zamora, Kassandra O. Rodriguez-Aguillón, Antonio Ovalle-Carcaño, Roberto Parra-Saldívar and Juan Eduardo Sosa-Hernández | Academic institution extensive, building-by-building wastewater-based surveillance platform for SARS-CoV-2 monitoring, clinical data correlation, and potential national proxy | Journal Article |
| Implementation | 2024 | T. L. Cowger, M. T. Sharp, J. D. Hart, B. O. Ojikutu, S. Nair and K. T. Hall | Implementation of Neighborhood-Level Wastewater-Based Epidemiology to Measure and Mitigate Inequities in SARS-CoV-2 Infection in Boston, Massachusetts | Journal Article |
| Implementation | 2024 | World Health Organization | Wastewater and environmental surveillance for one or more pathogens: Guidance on prioritization, implementation and integration | Repog |
| Implementation | 2023 | M. J. Swain, B. Carter, K. Snowdon and R. A. Faust | The Implementation and Utilization of Wastewater-Based Epidemiology: Experiences From a Local Health Department | Journal Article |
| Implementation | 2022 | Association of Public Health Laboratories | SARS-CoV-2 Wastewater Surveillance Testing Guide for Public Health Laboratories | Report |
| Implementation | 2022 | Meong Jin Joung, Chand S. Mangat, Edgard Mejia, Audra Nagasawa, Anil Nichani, Carolina Perez-Iracheta, Shelley W. Peterson and David Champredon | Coupling Wastewater-Based Epidemiological Surveillance and Modelling of SARS-COV-2/COVID-19: Practical Applications at the Public Health Agency of Canada | Journal Article |
| Implementation | 2021 | World Health Organization | SARS-CoV-2 genomic sequencing for public health goals | Report |
| Implementation, Economic | 2023 | Rebecca Fielding-Miller, Smruthi Karthikeyan, Tommi Gaines, Richard S. Garfein, Rodolfo A. Salido, Victor J. Cantu, Laura Kohn, Natasha K. Martin, Adriane Wynn, Carrissa Wijaya, Marlene Flores, Vinton Omaleki, Araz Majnoonian, Patricia Gonzalez-Zuniga, Megan Nguyen, Anh V. Vo, Tina Le, Dawn Duong, Ashkan Hassani, Samantha Tweeten, Kristen Jepsen, Benjamin Henson, Abbas Hakim, Amanda Birmingham, Peter De Hoff, Adam M. Mark, Chanond A. Nasamran, Sara Brin Rosenthal, Niema Moshiri, Kathleen M. Fisch, Greg Humphrey, Sawyer Farmer, Helena M. Tubb, Tommy Valles, Justin Morris, Jaeyoung Kang, Behnam Khaleghi, Colin Young, Ameen D. Akel, Sean Eilert, Justin Eno, Ken Curewitz, Louise C. Laurent, Tajana Rosing and Rob Knight | Wastewater and surface monitoring to detect COVID-19 in elementary school settings: The Safer at School Early Alert project | Journal Article |
| Implementation, Outcome | 2024 | C. Adams, M. Bias, R. M. Welsh, J. Webb, H. Reese, S. Delgado, J. Person, R. West, S. Shin and A. Kirby | The National Wastewater Surveillance System (NWSS): From inception to widespread coverage, 2020-2022, United States | Journal Article |
| Implementation, Outcome | 2024 | H. Brosky, S. M. Prasek, G. K. Innes, I. L. Pepper, J. Miranda, P. E. Brierley, S. L. Slinski, L. Polashenski, W. Q. Betancourt, K. Gronbach, D. Gomez, R. Neupane, J. Johnson, J. Weiss, H. D. Yaglom, D. M. Engelthaler, C. M. Hepp, K. Crank, D. Gerrity, J. R. Stewart and B. W. Schmitz | A framework for integrating wastewater-based epidemiology and public health | Journal Article |
| Implementation, Outcome | 2022 | David A. Larsen, Mary B. Collins, Qian Du, Dustin Hill, Tabassum Z. Insaf, Pruthvi Kilaru, Brittany L. Kmush, Frank Middleton, Abigail Stamm, Maxwell L. Wilder, Teng Zeng and Hyatt Green | Coupling freedom from disease principles and early warning from wastewater surveillance to improve health security | Journal Article |
| Implementation, Process, Impact | 2022 | M. Tlhagale, S. Liphadzi, J. Bhagwan, V. Naidoo, K. Jonas, L. van Vuuren, G. Medema, L. Andrews, F. Béen, M. L. Ferreira, A. M. Saatci, B. Alpaslan Kocamemi, F. Hassard, A. C. Singer, J. T. Bunce, J. M. S. Grimsley, M. Brown and D. L. Jones | Establishment of local wastewater-based surveillance programmes in response to the spread and infection of COVID-19 – case studies from South Africa, the Netherlands, Turkey and England | Journal Article |
| N/A | 2025 | Association of State and Territorial Health Officials | Framework for Addressing Ethical Considerations in Infectious Diseases Public Health Wastewater Surveillance | Report |
| N/A | 2025 | M. R. Arefin, C. Prouse, J. Wittmer, N. Amin, M. Assunção, A. Benezra, A. Chaudhuri, M. Diamond, S. Harshe, K. Hill-Tout, V. Koetz, D. Larsen, C. Mansfeldt, L. Melgaço, D. Nainani, A. V. Nair, C. C. Naughton, M. O'Donnell, C. Reimer, P. Robinson, J. Shelley and V. Srikantalah | Making Waves: A justice-centred framework for wastewater-based public health surveillance | Journal Article |
| N/A | 2024 | A. Mitra, J. A. Sefair, T. H. Grubesic, E. Helderop, J. R. Nelson, E. J. Bienenstock, A. Palladino, A. Valenti and J. Mertens | An optimization approach for biosurveillance in wastewater networks | Journal Article |
| N/A | 2024 | Africa CDC | Strengthening CrossBorder Surveillance and Information Sharing in Africa | Generic |
| N/A | 2024 | Global Consortium for Wastewater and Environmental Surveillance for Public Health | Towards the establishment of a Global Wastewater and Environmental Surveillance System for Public Health | Generic |
| N/A | 2024 | J. R. Mosher, J. E. Banta, R. Spencer-Hwang, C. C. Naughton, K. F. Kadonsky, T. Hile and R. G. Sinclair | An Environmental Equity Assessment Using a Social Vulnerability Index during the SARS-CoV-2 Pandemic for Siting of Wastewater-Based Epidemiology Locations in the United States | Journal Article |
| N/A | 2024 | K. Farkas, R. C. Williams, L. S. Hillary, A. Garcia-Delgado, E. Jameson, J. L. Kevill, M. J. Wade, J. M. S. Grimsley and D. L. Jones | Harnessing the Power of Next-Generation Sequencing in Wastewater-Based Epidemiology and Global Disease Surveillance | Journal Article |
| N/A | 2024 | Muhit Islam Emon, Yat Fei Cheung, James Stoll, Monjura Afrin Rumi, Connor Brown, Joung Min Choi, Nazifa Ahmed Moumi, Shafayat Ahmed, Haoqiu Song, Justin Sein, Shunyu Yao, Ahmad Khan, Suraj Gupta, Rutwik Kulkarni, Ali Butt, Peter Vikesland, Amy Pruden and Liqing Zhang | CIWARS: a web server for waterborne antibiotic resistance surveillance using longitudinal metagenomic data | Journal Article |
| N/A | 2024 | National Academies of Sciences, Engineering, and Medicine | Increasing the Utility of Wastewater-based Disease Surveillance for Public Health Action: A Phase 2 Report | Report |
| N/A | 2024 | R. Schneider, K. Weisbeck, K. Sheth, P. Sikes, K. Domakonda, L. Stadler, K. B. Ensor, R. Shaw, C. Berkobien, A. Wheeler, C. D. Johnson, C. Lengsfeld and L. Hopkins | Assessment of Public Health Agency and Utility Training Needs for CDC National Wastewater Surveillance System Jurisdictions in the United States | Journal Article |
| N/A | 2024 | U.S. Centers for Disease Control and Prevention | Developing a Wastewater Surveillance Sampling Strategy | Report |
| N/A | 2024 | V. M. Brown, E. A. Ogutu, A. E. Kauffman, S. S. Kennedy, R. A. Tenner, A. G. Wurcel, C. J. Zawitz, A. C. Spaulding and M. J. Akiyama | Guiding the Implementation of Wastewater-based Surveillance for Carceral Infection Control with Perspectives from People with Lived Experience of Incarceration during the COVID-19 Pandemic | Journal Article |
| N/A | 2024 | World Health Organization | Wastewater and Environmental Surveillance: Summary for Cholera | Report |
| N/A | 2024 | World Health Organization | Considerations for wastewater and environmental surveillance for monkeypox virus | Report |
| N/A | 2024 | World Health Organization | Wastewater and Environmental Surveillance: Summary for Poliovirus | Report |
| N/A | 2023 | A. Gholizadeh, M. Khiadani, M. Foroughi, H. Alizade Siuki and H. Mehrfar | Wastewater treatment plants: The missing link in global One-Health surveillance and management of antibiotic resistance | Journal Article |
| N/A | 2023 | A. Gitter, J. Oghuan, A. R. Godbole, C. A. Chavarria, C. Monserrat, T. Hu, Y. Wang, A. W. Maresso, B. M. Hanson, K. D. Mena and F. Q. Wu | Not a waste: Wastewater surveillance to enhance public health | Journal Article |
| N/A | 2023 | Andrew Bo Liu, Daniel Lee, Amogh Prabhav Jalihal, William P. Hanage and Michael Springer | Quantitatively assessing early detection strategies for mitigating COVID-19 and future pandemics | Journal Article |
| N/A | 2023 | Aparna Keshaviah, Megan B. Diamond, Matthew J. Wade, Samuel V. Scarpino, Warish Ahmed, Fabian Amman, Olusola Aruna, Andrei Badilla-Aguilar, Itay Bar-Or, Andreas Bergthaler, Julie E. Bines, Aaron W. Bivins, Alexandria B. Boehm, Jean-Martin Brault, Jean-Baptiste Burnet, Joanne R. Chapman, Angela Chaudhuri, Ana Maria de Roda Husman, Robert Delatolla, John J. Dennehy, Megan Beth Diamond, Celeste Donato, Erwin Duizer, Abiodun Egwuenu, Oran Erster, Despo Fatta-Kassinos, Aldo Gaggero, Deirdre F. Gilpin, Brent J. Gilpin, Tyson E. Graber, Christopher A. Green, Amanda Handley, Joanne Hewitt, Rochelle H. Holm, Heribert Insam, Marc C. Johnson, Rabia Johnson, Davey L. Jones, Timothy R. Julian, Asha Jyothi, Aparna Keshaviah, Tamar Kohn, Katrin G. Kuhn, Giuseppina La Rosa, Marie Lesenfants, Douglas G. Manuel, Patrick M. D'Aoust, Rudolf Markt, John W. McGrath, Gertjan Medema, Christine L. Moe, Indah Kartika Murni, Humood Naser, Colleen C. Naughton, Leslie Ogorzaly, Vicka Oktaria, Christoph Ort, Popi Karaolia, Ekta H. Patel, Steve Paterson, Mahbubur Rahman, Pablo Rivera-Navarro, Alex Robinson, Monica C. Santa-Maria, Samuel V. Scarpino, Heike Schmitt, Theodore Smith, Lauren B. Stadler, Jorgen Stassijns, Alberta Stenico, Renee A. Street, Elisabetta Suffredini, Zachary Susswein, Monica Trujillo, Matthew J. Wade, Marlene K. Wolfe, Habib Yakubu and Maria Ines Zanoli Sato | Wastewater monitoring can anchor global disease surveillance systems | Journal Article |
| N/A | 2023 | B. Kasprzyk-Hordern, F. Béen, L. Bijlsma, W. Brack, S. Castiglioni, A. Covaci, B. S. Martincigh, J. F. Mueller, A. L. N. van Nuijs, T. Oluseyi and K. V. Thomas | Wastewater-based epidemiology for the assessment of population exposure to chemicals: The need for integration with human biomonitoring for global One Health actions | Journal Article |
| N/A | 2023 | Colleen C. Naughton, Fernando A. Roman, Jr., Ana Grace F. Alvarado, Arianna Q. Tariqi, Matthew A. Deeming, Krystin F. Kadonsky, Kyle Bibby, Aaron Bivins, Gertjan Medema, Warish Ahmed, Panagis Katsivelis, Vajra Allan, Ryan Sinclair and Joan B. Rose | Show us the data: global COVID-19 wastewater monitoring efforts, equity, and gaps | Journal Article |
| N/A | 2023 | D. Parkins Michael, E. Lee Bonita, Nicole Acosta, Maria Bautista, R. J. Hubert Casey, E. Hrudey Steve, Kevin Frankowski and Xiao-Li Pang | Wastewater-based surveillance as a tool for public health action: SARS-CoV-2 and beyond | Journal Article |
| N/A | 2023 | Federica Armas, Franciscus Chandra, Wei Lin Lee, Xiaoqiong Gu, Hongjie Chen, Amy Xiao, Mats Leifels, Stefan Wuertz, Eric J. Alm and Janelle Thompson | Contextualizing Wastewater-Based surveillance in the COVID-19 vaccination era | Journal Article |
| N/A | 2023 | Guangquan Li, Hubert Denise, Peter Diggle, Jasmine Grimsley, Chris Holmes, Daniel James, Radka Jersakova, Callum Mole, George Nicholson, Camila Rangel Smith, Sylvia Richardson, William Rowe, Barry Rowlingson, Fatemeh Torabi, Matthew J. Wade and Marta Blangiardo | A spatio-temporal framework for modelling wastewater concentration during the COVID-19 pandemic | Journal Article |
| N/A | 2023 | J. Li, I. Hosegood, D. Powell, B. Tscharke, J. Lawler, K. V. Thomas and J. F. Mueller | A global aircraft-based wastewater genomic surveillance network for early warning of future pandemics | Journal Article |
| N/A | 2023 | Joseph W. Shingleton, Chris J. Lilley and Matthew J. Wade | Evaluating the theoretical performance of aircraft wastewater monitoring as a tool for SARS-CoV-2 surveillance | Journal Article |
| N/A | 2023 | National Academies of Sciences, Engineering, and Medicine | Wastewater-based Disease Surveillance for Public Health Action | Report |
| N/A | 2023 | Pengbo Liu, Lizheng Guo, Matthew Cavallo, Caleb Cantrell, Stephen Patrick Hilton, Jillian Dunbar, Robbie Barbero, Robert Barclay, Orlando Sablon, III, Marlene Wolfe, Ben Lepene and Christine Moe | Evaluation of Simple and Convenient Methods for SARS-CoV-2 Detection in Wastewater in high and Low Resource Settings | Journal Article |
| N/A | 2023 | Rasha Maal-Bared, Kari Brisolara, Mark Knight and Cresten Mansfeldt | To sample or not to sample: A governance-focused decision tree for wastewater service providers considering participation in wastewater-based epidemiology (WBE) in support of public health programs | Journal Article |
| N/A | 2023 | Sana Zakaria | Testing the Waters: Using Wastewater Surveillance as an Early Warning System | Commentary |
| N/A | 2023 | T. B. Henriques, S. T. Cassini and R. de Pinho Keller | Contribution of wastewater-based epidemiology to SARS-CoV-2 screening in Brazil and the United States | Journal Article |
| N/A | 2023 | T. Prado, G. Rey-Benito, M. P. Miagostovich, M. I. Z. Sato, V. B. Rajal, C. R. Mota, A. D. Pereira, M. R. F. Barbosa, C. F. Mannarino and A. S. da Silva | Wastewater-based epidemiology for preventing outbreaks and epidemics in Latin America - Lessons from the past and a look to the future | Journal Article |
| N/A | 2023 | WHO Regional Office for South-East Asia | Strategic Framework for Action for Strengthening Surveillance, Risk Assessment and Field Epidemiology for Health Security Threats in the WHO South-East Asia Region | Report |
| N/A | 2023 | Xindi C. Hu, Stacie K. Reckling and Aparna Keshaviah | Assessing health equity in wastewater monitoring programs: Differences in the demographics and social vulnerability of sewered and unsewered populations across North Carolina | Journal Article |
| N/A | 2022 | D. Cantillon and A. P. Roberts | Development and evaluation of TaqMan-based, one-step, real-time RT-PCR assays for pepper mild mottle virus detection for near source tracking and wastewater-based epidemiology validation | Journal Article |
| N/A | 2022 | Doug Manuel, Carlo Alberto Amadei, Jonathon R. Campbell, Jean-Martin Brault, Amy Zierler and Jeremy Veillard | Strengthening Public Health Surveillance Through Wastewater Testing: An Essential Investment for the COVID-19 Pandemic and Future Health Threats | Report |
| N/A | 2022 | M. Petala, M. Kostoglou, Th Karapantsios, C. I. Dovas, Th Lytras, D. Paraskevis, E. Roilides, A. Koutsolioutsou-Benaki, G. Panagiotakopoulos, V. Sypsa, S. Metallidis, A. Papa, E. Stylianidis, A. Papadopoulos, S. Tsiodras and N. Papaioannou | Relating SARS-CoV-2 shedding rate in wastewater to daily positive tests data: A consistent model based approach | Journal Article |
| N/A | 2022 | Natalie Ram, Lance Gable and Jeffrey L. Ram | The Future of Wastewater Monitoring for the Public Health | Law Review |
| N/A | 2022 | Pruthvi Kilaru, Dustin Hill, Kathryn Anderson, Mary B Collins, Hyatt Green, Brittany L Kmush and David A Larsen | Wastewater Surveillance for Infectious Disease: A Systematic Review | Journal Article |
| N/A | 2022 | S. E. Hrudey and B. Conant | The devil is in the details: emerging insights on the relevance of wastewater surveillance for SARS-CoV-2 to public health | Journal Article |
| N/A | 2021 | A. E. Kirby, M. S. Walters, W. C. Jennings, R. Fugitt, N. LaCross, M. Mattioli, Z. A. Marsh, V. A. Roberts, J. W. Mercante, J. Yoder and V. R. Hill | Using Wastewater Surveillance Data to Support the COVID-19 Response - United States, 2020-2021 | Journal Article |
| N/A | 2021 | A. Pruden, P. J. Vikesland, B. C. Davis and A. M. de Roda Husman | Seizing the moment: now is the time for integrated global surveillance of antimicrobial resistance in wastewater environments | Journal Article |
| N/A | 2021 | Aparna Keshaviah, C. Hu Xindi and Marisa Henry | Developing a Flexible National Wastewater Surveillance System for COVID-19 and Beyond | Journal Article |
| N/A | 2021 | Jill McClary-Gutierrez, Mia Mattioli, Perrine Marcenac, Andrea Silverman, Alexandria Boehm, Kyle Bibby, Michael Balliet, Francis de los Reyes, Daniel Gerrity, John Griffith, Patricia Holden, Dimitrios Katehis, Greg Kester, Nathan LaCross, Erin Lipp, Jonathan Meiman, Rachel Noble, Dominique Brossard and Sandra McLellan | SARS-CoV-2 Wastewater Surveillance for Public Health Action | Journal Article |
| N/A | 2021 | Juliette O’Keeffe | Wastewater-based epidemiology: current uses and future opportunities as a public health surveillance tool | Journal Article |
| N/A | 2021 | K. Hill, A. Zamyadi, D. Deere, P. A. Vanrolleghem and N. D. Crosbie | SARS-CoV-2 known and unknowns, implications for the water sector and wastewater-based epidemiology to support national responses worldwide: early review of global experiences with the COVID-19 pandemic | Journal Article |
| N/A | 2021 | S. E. Déglin, C. L. Chen, D. J. Miller, R. J. Lewis, E. T. Chang, A. K. Hamade and H. S. Erickson | Environmental epidemiology and risk assessment: Exploring a path to increased confidence in public health decision-making | Journal Article |
| N/A | 2021 | T. Dharmadhikari, R. Yadav, S. Dastager and M. Dharne | Translating SARS-CoV-2 wastewater-based epidemiology for prioritizing mass vaccination: a strategic overview | Journal Article |
| N/A | 2021 | World Health Organization | Genomic sequencing of SARS-CoV-2: a guide for implementation for maximum impact on public health | Report |
| N/A | 2020 | Alshehri Ali, Owied Al Moteri Modi, Plummer. Virginia, Ruth Endacott and Abdulellah Al Thobaity | Using Logic Model and Key Performance Indicators to Construct A Field Hospital for Coronavirus Pandemic Cases | Journal Article |
| N/A | 2020 | G. Medema, F. Been, L. Heijnen and S. Petterson | Implementation of environmental surveillance for SARS-CoV-2 virus to support public health decisions: Opportunities and challenges | Journal Article |
| N/A | 2020 | Natalie Sims and Barbara Kasprzyk-Hordern | Future perspectives of wastewater-based epidemiology: Monitoring infectious disease spread and resistance to the community level | Journal Article |
| Outcome | 2024 | A. White, G. Iverson, L. Wright, J. T. Fallon, 3rd, K. P. Briley, C. Yin, W. Huang and C. Humphrey | Wastewater based epidemiology as a surveillance tool during the current COVID-19 pandemic on a college campus (East Carolina University) and its accuracy in predicting SARS-CoV-2 outbreaks in dormitories | Journal Article |
| Outcome | 2024 | Alexander Yu, Elisabeth Burnor, Angela Rabe, Sarah Rutschmann, Marlene Wolfe, Jessie Burmester, Chao-Yang Pan, Alice Chen, Hugo Guevara, Christina Morales, Debra Wadford, Alexandria Boehm and Duc Vugia | Wastewater Surveillance for Norovirus, California, USA | Journal Article |
| Outcome | 2024 | B. Baguune, E. B. Laryea, J. A. Frimpong, S. Dapaa, K. K. Achempem, E. Kenu and D. O. Laryea | Evaluation of the environmental polio surveillance system-Northern Region, Ghana, 2021 | Journal Article |
| Outcome | 2024 | Binod Pant, Salman Safdar, Calistus N. Ngonghala and Abba B. Gumel | Mathematical assessment of wastewater-based epidemiology to predict SARS-CoV-2 cases and hospitalizations in Miami-Dade County | Journal Article |
| Outcome | 2024 | Erin Whitehouse, Nancy Gerloff, Randall English, Stacie Reckling, Mohammed Alazawi, Meghan Fuschino, Kirsten St George, Daniel Lang, Eli Rosenberg, Enoma Omoregie, Jennifer Rosen, Alyse Kitter, Colin Korban, Massimo Pacilli, Trisha Jeon, Joseph Coyle, Russell Faust, Irene Xagoraraki, Brijen Miyani, Charles Williams, James Wendt, Sarah Owens, Rosemarie Wilton, Rachel Poretsky, Lynn Sosa, Kathy Kudish, Manisha Juthani, Elizabeth Zaremski, Susan Kehler, Nagla Bayoumi and Sarah Kidd | Wastewater Surveillance for Poliovirus in Selected Jurisdictions, United States, 2022–2023 | Journal Article |
| Outcome | 2024 | Hannes Schenk, Wolfgang Rauch, Alessandro Zulli and Alexandria B. Boehm | SARS-CoV-2 surveillance in US wastewater: Leading indicators and data variability analysis in 2023–2024 | Journal Article |
| Outcome | 2024 | R. Schneider, K. Domakonda, S. Bhandari, L. Stadler, K. B. Ensor, A. Mulenga, C. D. Johnson and L. Hopkins | Implementing an Alert System for Communicating Actionable Wastewater Surveillance Results to School Communities, Houston, Texas, 2023-2024 | Journal Article |
| Outcome | 2023 | M. Khan, L. Li, L. Haak, S. H. Payen, M. Carine, K. Adhikari, T. Uppal, P. D. Hartley, H. Vasquez-Gross, J. Petereit, S. C. Verma and K. Pagilla | Significance of wastewater surveillance in detecting the prevalence of SARS-CoV-2 variants and other respiratory viruses in the community - A multi-site evaluation | Journal Article |
| Outcome | 2023 | N. Trigo-Tasende, J. A. Vallejo, S. Rumbo-Feal, K. Conde-Pérez, M. Vaamonde, Á López-Oriona, I. Barbeito, M. Nasser-Ali, R. Reif, B. K. Rodiño-Janeiro, E. Fernández-Álvarez, I. Iglesias-Corrás, B. Freire, J. Tarrío-Saavedra, L. Tomás, P. Gallego-García, D. Posada, G. Bou, I. López-de-Ullibarri, R. Cao, S. Ladra and M. Poza | Wastewater early warning system for SARS-CoV-2 outbreaks and variants in a Coruña, Spain | Journal Article |
| Outcome | 2023 | X. T. Shao, S. Y. Liu, Y. T. Zhao, B. Jiang, J. G. Lin and D. G. Wang | Evaluation of eight psychoactive drugs used in Chinese cities by wastewater-based epidemiology | Journal Article |
| Outcome | 2022 | Avian White | Evaluation of the use of Wastewater Based Epidemiology as a Surveillance Tool and the Potential effects of Vaccines and Students Beliefs and Practices in Mitigating the Spread of COVID-19 Among Students at East Carolina University | Dissertation |
| Outcome | 2022 | M. Landstrom, E. Braun, E. Larson, M. Miller and G. H. Holm | Efficacy of SARS-CoV-2 wastewater surveillance for detection of COVID-19 at a residential private college | Journal Article |
| Outcome | 2021 | E. C. Rouchka, J. H. Chariker, K. Saurabh, S. Waigel, W. Zacharias, M. Zhang, D. Talley, I. Santisteban, M. Puccio, S. Moyer, R. H. Holm, R. A. Yeager, K. J. Sokoloski, J. Fuqua, A. Bhatnagar and T. Smith | The Rapid Assessment of Aggregated Wastewater Samples for Genomic Surveillance of SARS-CoV-2 on a City-Wide Scale | Journal Article |
| Outcome | 2018 | A. Garg, S. Pattamadilok and S. Bahl | Successes and challenges of expansion of environmental poliovirus surveillance in the WHO South-East Asia Region | Journal Article |
| Outcome | 2016 | T. J. Muluh, A. W. Hamisu, K. Craig, P. Mkanda, E. Andrew, J. Adeniji, A. Akande, A. Musa, I. Ayodeji, G. Nicksy, R. Banda, S. G. Tegegne, P. Nsubuga, A. Oyetunji, O. Diop, R. G. Vaz and A. J. G. Muhammad | Contribution of Environmental Surveillance Toward Interruption of Poliovirus Transmission in Nigeria, 2012-2015 | Journal Article |
| Outcome, Economic | 2023 | D. A. Bowes, E. M. Driver, S. Savic, Q. W. Cheng, C. M. Whisner, R. Krajmalnik-Brown and R. U. Halden | Integrated multiomic wastewater-based epidemiology can elucidate population-level dietary behaviour and inform public health nutrition assessments | Journal Article |
| Outcome, Economic | 2022 | S. Ali, E. K. Gudina, A. Gize, A. Aliy, B. T. Adankie, W. Tsegaye, G. B. Hundie, M. B. Muleta, T. R. Chibssa, R. Belaineh, D. Negessu, D. Shegu, G. Froeschl and A. Wieser | Community Wastewater-Based Surveillance Can Be a Cost-Effective Approach to Track COVID-19 Outbreak in Low-Resource Settings: Feasibility Assessment for Ethiopia Context | Journal Article |
| Outcome, Formative | 2021 | H. J. Majeed, M. V. Riquelme, B. C. Davis, S. Gupta, L. Angeles, D. S. Aga, E. Garner, A. Pruden and P. J. Vikesland | Evaluation of Metagenomic-Enabled Antibiotic Resistance Surveillance at a Conventional Wastewater Treatment Plant | Journal Article |
| Outcome, Impact, Economic | 2024 | J. Sanjak, E. McAuley, J. Raybern, R. Pinkham, J. Tarnowski, N. Miko, B. Rasmussen, C. Manalo, M. Goodson, B. Stamps, B. Necciai, S. Sozhamannan and E. Maier | Wastewater Surveillance Pilot at US Military Installations: Cost Model Analysis | Journal Article |
| Process | 2024 | Douglas G. Manuel, Carol Bennett, Emma Brown, David L. Buckeridge, Yoni Freedhoff, Sarah Funnell, Farah Ishtiaq, Matthew J. Wade, David Moher and Phes- E. F. Executive Group the | Developing an evaluation framework for public health environmental surveillance: Protocol for an international, multidisciplinary e-Delphi study | Journal Article |
| Process | 2022 | Beatrice Daleiden, Harald Niederstätter, Martin Steinlechner, Stefan Wildt, Manfred Kaiser, Cornelia Lass-Flörl, Wilfried Posch, Stefan Fuchs, Bernhard Pfeifer, Andreas Huber and Herbert Oberacher | Wastewater surveillance of SARS-CoV-2 in Austria: development, implementation, and operation of the Tyrolean wastewater monitoring program | Journal Article |
| Process, Outcome | 2024 | Public Health Scotland | Evaluating the public health utility of wastewater-based surveillance of SARS-CoV-2 in Scotland: Technical Report | Report |
